# Impact of a Healthy Dietary Pattern on Gut Microbiota and Systemic Inflammation in Humans

**DOI:** 10.3390/nu10111783

**Published:** 2018-11-16

**Authors:** Vibeke H. Telle-Hansen, Kirsten B. Holven, Stine M. Ulven

**Affiliations:** 1 Faculty of Health Sciences, Oslo Metropolitan University, P.O. Box 4, St. Olavsplass, 0130 Oslo, Norway; vtelle@oslomet.no; 2 Department of Nutrition, Institute of Basic Medical Sciences, Faculty of Medicine, University of Oslo, P.O. Box 1046, Blindern, 0317 Oslo, Norway; kirstenholven@medisin.uio.no; 3 National Advisory Unit on Familial Hypercholesterolemia, Department of Endocrinology, Morbid Obesity and Preventive Medicine, Oslo University Hospital, Rikshospitalet, P.O. Box 4950, Nydalen, 0424 Oslo, Norway

**Keywords:** gut microbiota, inflammation, diet, humans, intervention studies

## Abstract

Gut microbiota have recently been suggested to play a part in low-grade systemic inflammation, which is considered a key risk factor for cardiometabolic disorders. Diet is known to affect gut microbiota; however, the effects of diet and dietary components on gut microbiota and inflammation are not fully understood. In the present review, we summarize recent research on human dietary intervention studies, investigating the effects of healthy diets or dietary components on gut microbiota and systemic inflammation. We included 18 studies that reported how different dietary components altered gut microbiota composition, short-chain fatty acid levels, and/or inflammatory markers. However, the heterogeneity among the intervention studies makes it difficult to conclude whether diets or dietary components affect gut microbiota homeostasis and inflammation. More appropriately designed studies are needed to better understand the effects of diet on the gut microbiota, systemic inflammation, and risk of cardiometabolic disorders.

## 1. Introduction

**Figure 1:**
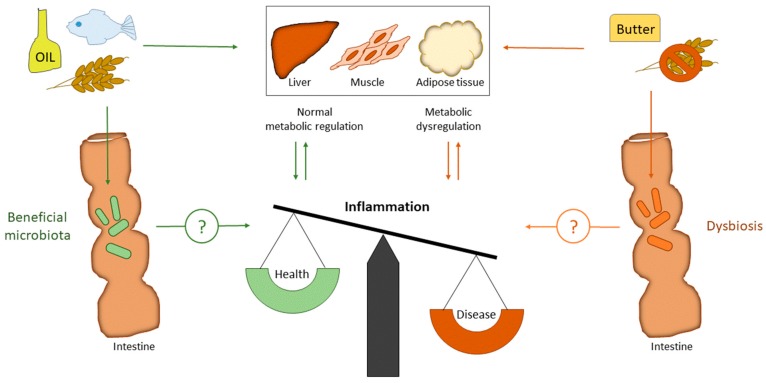
Impact of dietary fat (SFA versus PUFA) and fiber on gut microbiota and metabolic regulation. SFA is well known to induce metabolic dysregulation, while PUFA and fiber are well known to improve metabolic regulation. High intake of SFA will increase the cholesterol level in blood, which is an important risk factor for CVD. PUFA and fiber will decrease blood cholesterol and triglycerides, in addition to improve blood glucose regulation. The underlying mechanism of cardiometabolic disorders is inflammation. Both fiber and fat may change the gut microbiota composition. However, whether gut microbiota regulate inflammation directly by dietary interventions in humans is still to be elucidated. CVD: Cardiovascular diseases; PUFA: polyunsaturated fatty acids; SFA: saturated fatty acids.

Cardiometabolic disorders such as type 2 diabetes (T2D), cardiovascular diseases (CVD), and obesity are major health concerns. The underlying mechanism of these diseases is chronic low-grade systemic inflammation, and strategies to dampen inflammation are of great therapeutic interest [1]. Several studies suggest that there is an interaction between the gut microbiota and the immune system, and that changes in gut microbiota may contribute to chronic inflammation [2]. Furthermore, there is accumulating evidence indicating that an altered gut microbiota may contribute to the development of metabolic dysfunction by affecting risk factors such as insulin sensitivity and glucose metabolism [3] (Figure 1).

Gut microbiota have evolved with humans through a symbiotic relationship, which provides many benefits for humans, including protecting against pathogens, training the immune system, maintaining intestinal barrier integrity, contributing nutrients, such as vitamins (vitamin K and B), and producing short-chain fatty acids (SCFAs) [4,5]. Even though anaerobic bacteria dominate, the gut microbiota include members from all three domains of life (Bacteria, Archaea, and Eukarya) [5]. The major bacteria species found in the human gut, which make up more than 90% of the gut microbiota, belong to one of two bacteria phyla: *Firmicutes* and *Bacteroidetes* [5,6,7,8,9]. Some of the members of the *Firmicutes* and *Bacteroidetes* phyla are shown in Table 1. A complete overview of the bacteria in the human gastrointestinal tract is given by others [10].

The gut microbiota composition changes throughout the life course [9], and several environmental factors, including diet, may modulate the composition [9,12]. A modulation may lead to a more unbeneficial microbiota composition associated with diseases or dysfunction known as microbiota dysbiosis [13,14]. Dysbiosis is characterized by reduced bacterial diversity and altered composition, and is associated with cardiometabolic disorders like T2D and obesity [13,14]. Dysbiosis may affect gut permeability, and the low-grade chronic inflammation observed in cardiometabolic disorders may be triggered by impaired gut permeability and subsequently increased circulating levels of lipopolysaccharides (LPS) [2,11,15]. Furthermore, subjects with low bacterial diversity are characterized by increased low-grade inflammation when compared to subjects with high bacterial diversity [16]. SCFAs, mainly acetate, propionate, and butyrate, are produced by the gut microbiota through fermentation of indigestible fiber and have been suggested to have beneficial cardiometabolic effects [5]. SCFAs, in particular butyrate, are used as energy by intestinal epithelium cells to help strengthen intestinal barrier integrity, thereby inhibiting the ability of microbial molecules, including LPS, to stimulate the immune system and induce inflammation [17,18,19]. The underlying molecular mechanisms that account for the microbiota‒immune system interaction are not completely understood but SCFAs are known to modulate several cellular processes including gene expression, chemotaxis, proliferation, differentiation, and apoptosis [19].

In order to prevent cardiometabolic disorders, it is important to better understand the role of diet on gut microbiota. Food with added bacteria (probiotics) has been investigated for possible beneficial health effects. To what degree probiotics may have a beneficial health effect is debated and has been reviewed elsewhere [10,20]. Soluble dietary fiber is well known for having beneficial cardiometabolic effects by reducing cholesterol levels, controlling blood glucose, and regulating body weight [21,22]. Traditionally, the mechanisms have been attributed to a viscous gel formed in the stomach and intestine after soluble fiber intake [23]. The gel inhibits and slows down the absorption of carbohydrates and reabsorption of bile acids, which leads to increased synthesis of bile acids from cholesterol in the liver [23,24,25]. Dietary fiber is fermented by the gut microbiota into SCFAs, and, therefore, the beneficial effects of dietary fiber most likely include gut microbiota. One of the first studies investigating dietary effects on gut microbiota compared the gut microbiota in children aged 1‒6 years between an African population in Burkina Faso that consumed a traditional, rural diet high in fiber and low in fat, with a European population from Italy that consumed a modern diet high in fat and low in fiber [26]. The African children had increased bacterial diversity and higher levels of *Bacteroidetes* and SCFAs in feces compared with the Italian children, who had low diversity and a higher amount of fecal *Firmicutes* and a lower amount of fecal SCFAs [26]. Similar findings have been reported by others [27], and it is generally assumed that a low-fat/high-fiber diet will result in increased bacteria diversity compared with a high-fat/low-fiber diet.

Although prebiotic soluble fibers and probiotics are the most studied dietary factors associated with gut microbiota changes, other nutrients and different dietary patterns may also affect gut microbiota. There is strong evidence that replacing SFA with PUFA will reduce the risk of CVD [28] by reducing plasma LDL cholesterol, which is an established risk factor of CVD [29]. Studies suggest that not only the total amount of fat but also the type of fat may affect metabolic regulation through altered microbiota homeostasis [30]. Traditionally, it has been assumed that the digestion and absorption of dietary fat mainly takes place in the small intestine, while only a small portion reaches the colon [12]. However, recent findings by Gabert et al. [30] have shown that about 7% of 13C labeled dietary fat was excreted in the feces of healthy subjects, showing that a significant amount of dietary fat passes from the small intestine to the colon [11]. Caesar and colleagues compared the effects of fish oil, high in polyunsaturated fatty acids (PUFAs), or lard, high in saturated fatty acids (SFAs), on the microbiota in mice. In contrast to mice given PUFAs, the mice given SFA changed their gut microbiota composition, became obese, and showed evidence of adipose tissue inflammation. Fecal transplantation from mice given PUFA to those given SFA were sufficient to reverse inflammation [31]. Mice on a high-fat diet have increased adipose tissue and low-grade inflammation due to elevated LPS levels, whereas LPS-infected mice show similar metabolic changes to mice given a high-fat diet [32]. 

To what degree diet and dietary components, including fiber and fat, change the gut microbiota and affect circulating inflammatory markers in humans remains to be elucidated. The aim of the present review was to summarize recent human intervention studies investigating the effect of diet or dietary components on gut microbiota and systemic inflammation.

## 2. Materials and Methods

To identify relevant studies, we performed a literature search in PubMed in May 2018. Only original articles with interventional trials in humans were included. Furthermore, only studies with information about gut microbiota and/or SCFA, inflammatory markers and foods or whole diets were included. It is well known that nutrients and diets affect human health and that an unhealthy diet is one of the main risk factors for metabolic diseases. We particularly focused on the degree to which nutrients, alone or as part of a healthy diet, may affect the bacteria in the gut. Interventions with added probiotics or nutraceuticals were therefore not included. Inflammatory markers were defined as pro-inflammatory cytokines, acute-phase proteins, adhesion molecules, chemokines and calprotectin, LPS or LPS-binding protein (LBP). The search terms were microbiota AND inflammation AND dietary fat AND/OR dietary fiber AND/OR Mediterranean diet AND/OR Nordic diet AND/OR Western diet, giving 564 articles. We included studies that fulfilled the following criteria: gut microbiota and dietary interventions, and at least one inflammatory marker measured. The studies were included independent of primary and secondary outcomes. Moreover, we excluded studies that fulfilled at least one of the following criteria: not an original study (for example, an editorial, review, or conference paper), cohort study, cross-sectional study, animal study, articles not written in English, or lack of inclusion criteria measurements (as defined previously). Duplicate articles were removed. In total, 18 articles were reviewed in full text and included in the present article.

## 3. Results and Discussion

In the present review, we summarize and discuss the effects of diet and dietary components on gut microbiota and markers of inflammation in human studies, from a total of 18 post-prandial, and short- or long-term intervention trials (parallel and crossover design; three weeks to six months). Half of the included studies investigated the effect of fiber or wholegrain (nine of 18 articles) [33,34,35,36,37,38,39,40,41], while the rest of the articles investigated the effect of dietary fat, other dietary components or dietary patterns (two [42,43], three [44,45,46] and four [47,48,49,50] articles, respectively), on gut microbiota and inflammatory markers. The study populations were very heterogeneous among the studies, and included both males and females, adults and children, healthy subjects or subjects with chronic diseases, normal weight, overweight and obese subjects. The heterogeneity in study design, study subjects, type of dietary intervention, different measurements of inflammatory markers, lack of standardized reporting at taxonomic level and in regards to gut microbiota composition made it impossible to draw a firm conclusion about the effect of dietary components on microbiota and inflammation. The present review gives an updated overview of the field and the state of knowledge.

### 3.1. Dietary Fiber and Whole Grains

Vanegas et al. performed a six-week intervention with whole or refined grains, and measured inflammatory responses, gut microbiota, and microbial products in 81 healthy adults [33]. The authors reported a significant increase in *Lachnospira* (SCFA producer) and a decrease in *Enterobacteriaceae* (acting pro-inflammatory). However, there were no significant changes in stool or plasma cytokines (tumor necrosis factor alpha (TNFα), interleukin 6 (IL-6), IL-8, IL-1β, IL-17, interferon gamma (IFNγ), transforming growth factor beta (TGFβ), LPS-binding protein (LBP)) [33] (Table 2). In another study, plasma SCFAs and inflammatory marker levels were determined following a 12-week intervention with either whole-grain or refined cereal foods in 54 adults with metabolic syndrome (MetS) [39]. Both intervention groups had the same energy intake, nutrient composition and main carbohydrate source. Whole-grain food intervention resulted in significantly increased levels of plasma propionate compared with the control group, but no significant differences in high-sensitivity C-reactive protein (hsCRP), IL-6, IL-1RA, and TNFα levels were observed [39] (Table 3). Canfora et al. investigated the effects of supplementation with galacto-oligosaccharides (GOS) on human gut microbiota composition and metabolism in a 12-week intervention with 44 prediabetic overweight/obese subjects [41]. GOS supplementation led to a significant increase in *Bifidobacterium* species, *Prevotella oralis et rel.*; *Prevotella melaninogenica et rel.*; *Bacteroides stercoris et rel.*; and *Sutterella wadsworthia et rel.* in feces, compared with the control group. However, microbial richness or diversity was unaffected. No differences in the levels of SCFAs, LBP, or other markers of inflammation (IL-6, IL-8, TNFα) were seen between the groups [41] (Table 3). Nicolucci et al. investigated the effect of prebiotics (oligofructose-enriched inulin) or maltodextrin (control) on body composition, markers of inflammation, and composition of the intestinal microbiota in children (7‒12 years) with overweight or obesity during a 16-week intervention [35]. Children consuming prebiotics had a significant decrease in plasma IL-6, and a significant increase in *Bifidobacterium* spp compared with controls. A significant within-group increase in *Actinobacteria*, *Bifidobacterium spp*, *Collinsella* and decrease in *Ruminococcus* after intervention with oligofructose-enriched inulin was reported. Moreover, a reduction in *Clostridium XVIII*, *Actinomyces*, *Dorea*, and *Eggerthella* within the control group was also observed [35] (Table 2). Nilsson et al. evaluated the modulatory effects of barley in combination with probiotics on markers of metabolic regulation in three four-day intervention periods where barley kernel bread or white wheat flour bread was included in the normal diet of 21 healthy subjects [36]. There were no significant differences in inflammatory markers after intake of barley bread compared with white bread. Barley bread with and without probiotics significantly increased breath H^2^ (measure of gut bacteria activity), compared with intake of white bread, whereas barley bread with probiotics significantly increased the serum level of plasminogen activator inhibitor (sPAI-1) after the standardized breakfast compared with intake of both white bread and barley bread without probiotics [36] (Table 2). An impaired intestinal barrier is hypothesized to increase the translocation of the Gram-negative bacteria cell membrane component LPS into the circulation, which results in metabolic endotoxemia and low-grade inflammation [51]. In a study by Pedersen et al., they investigated the link between intestinal permeability and intestinal bacteria in 29 men with T2D who were randomized to a prebiotic (galacto-oligosaccharide mixture) or control (maltodextrin) supplement for 12 weeks [40]. There was no effect of prebiotic fiber on inflammatory markers (LPS, LBP, hsCRP, CD14, IL-6, TNFα) or bacterial abundance, diversity, and richness; however, changes in the bacterial family *Veillonellaceae* were significantly inversely correlated with changes in IL-6 levels within the prebiotic group [40] (Table 3). Morales et al. investigated the effect of prebiotic oligofructose in combination with orlistat on the colonic ecosystem in 41 healthy volunteers during a one-week intervention [34]. No change in gut barrier function was observed; however, an increase in *Bifidobacterium* within the Orlistat‒prebiotic group was observed. There were no changes in hsCRP, IL-6, or calprotectin between the groups, but hsCRP significantly decreased within the Orlistat‒prebiotic group, IL-6 decreased within the Orlistat, prebiotic, and control groups, and calprotectin increased within the Orlistat group. Fecal SCFAs remained unchanged, except for a significant decrease in isobutyrate and isovalerate within the Orlista‒prebiotic group [34] (Table 2). Crohn’s disease is a chronic inflammatory condition of the gastrointestinal tract, associated with dysbiosis. In a four-week randomized, double-blind, placebo-controlled trial with 103 patients with Crohn’s disease given 15 g/day of fructo-oligosaccharides (FOS) or maltodextrin (control), there was a significant reduction in IL-6 positive dendritic cells compared with the control group [37]. There were no significant changes in gut microbiota (*Bifidobacteria* and *Faecalibacterium prausnitzii*) or IL-10 and IL-12p40 dendritic cells between groups, but there was a significant increase in IL-10 dendritic cells within the FOS group [37] (Table 3). The lack of effect on gut microbiota is in contrast to previous studies in healthy subjects where 10–15 g of prebiotics per day increased fecal *Bifidobacteria* and *Faecalibacterium prausnitzii* [52,53]. Vulevic et al. studied the effects of a galactooligosaccaride mixture (B-GOS) on gut microbiota and immune function in 45 overweight/obese adults with increased risk of MetS in a double-blind, randomized, placebo (maltodextrine)-controlled crossover study [38]. Intervention with B-GOS significantly increased the number of fecal *Bifidobacterium spp*., and decreased *Bacteroides spp.*, *C. histolyticum* group, and *Desulfovibrio spp.* compared with the control group. Furthermore, fecal calprotectin and plasma CRP decreased after FOS intervention compared to the control group [38] (Table 3). There were no significant differences in granulocytes colony-stimulating factor (G-CSF), IL-6, IL-10, IL-8, and TNFα levels between the groups during the intervention.

Diet and fiber have been shown to effectively change gut microbiota, as early as 1‒3 days after an intervention [54]. Although most studies (six out of nine) in this review reported changes in gut microbiota composition between control and intervention groups, three studies did not [34,37,40]. Even though the causal relationship between gut microbiota and chronic diseases is unclear, some bacteria have been suggested to be more beneficial, like the *Bifidobacteria* and *Firmicutes*, while others, like *Bacteroidetes*, have been linked to increased risk of diseases [55,56]. However, the association between bacteria species and different diseases is not consistent among studies [57,58,59]. A study by Canfora and co-workers found increased Prevotella (a genus in the *Firmicutes* phylum) in prediabetic subjects after intervention with GOS [41], indicating improved health. However, Bacteroides (a genus of the *Bacteroidetes* phylum) also increased, indicating a possible negative health effect, but no changes in inflammatory markers were observed [41]. A reduction in *Bifidobacteria* has been linked to obesity and T2D [60]. In this review, two of the studies found increased levels of *Bifidobacteria* in subjects with obesity and MetS [38] or prediabetes [41], indicating an improved gut microbiota profile after a dietary intervention. However, only the study with subjects with MetS found a reduction in pro-inflammatory IL-6 levels [38]. Three of the nine studies found that consuming fiber significantly decreased inflammatory markers (IL-6, CRP or calprotectin) compared with the placebo groups [35,37,38], while another study found that changes in the bacterial family *Veillonellaceae* correlated inversely with changes in IL-6 levels after intake of fiber [40]. In contrast to these findings, Vanegas et al. and Nilsson et al. found an increase in inflammatory markers (TNFα and sPAI-1) after fiber intake in healthy adults [33,36]. Almost half of the studies (four out of nine) found no effect on inflammatory markers after intake of fiber or whole grains compared with placebo. The disparity among the currently available studies suggests that better designed randomized controlled dietary interventional studies are needed before firm conclusions on whether the intake of fiber leads to a beneficial change in bacterial composition, and a reduction in low-grade inflammation among healthy subjects but also among those with metabolic diseases.

### 3.2. Dietary Fat

Martín-Peláez et al. investigated the effect of olive oil with three different concentrations of polyphenols (80 mg, 500 mg, and 500 mg + thyme, respectively) for three weeks on gut microbiota and inflammatory markers in 10 hypercholesterolemic adults [42]. The number of total bacteria was significantly reduced in the olive oil group, with the lowest concentration of polyphenols (80 mg) compared with those who consumed olive oil with the highest concentration (500 mg). There was no significant change in the ratio of *Firmicutes*/*Bacteroidetes*, IgA-coated bacteria, or IgA in feces between the groups. However, within the group that consumed olive oil with 500 mg polyphenols, there was a significant increase in IgA-coated bacteria. Plasma levels of CRP were also significantly increased in this group compared to the other groups. No significant differences in the fecal levels of TNFα, IL-6, and calprotectin were found between the groups [42] (Table 3). In another study where 35 T2D patients were given either a standard diet (control) or 100 g of sardines equaling about 3 g EPA + DHA per day for six months [43], both the sardine group and the control group significantly decreased the amount of *Firmicutes* and increased *E. coli* concentrations. However, only the sardine group had significantly decreased *Firmicutes*/*Bacteroidetes* ratio and increased *Bacteroides‒Prevotella* group compared with the baseline. There were no significant changes in CRP, IL-6, IL-10, and TNFα levels between the groups after the intervention [43] (Table 3).

Two of the studies in this review investigated the effects of dietary fat on gut microbiota and inflammation, through the consumption of either fish rich in omega-3 fatty acids or olive oil. Both studies were performed in subjects with chronic diseases. The studies found changes in gut microbiota after the intervention with dietary fat, but no changes in inflammatory markers. Studies in mice and humans have linked an increase in the *Firmicutes*/*Bacteroidetes* ratio to metabolic dysregulation, such as obesity and insulin resistance [61,62]. A study by Balfego et al. found a decreased *Firmicutes*/*Bacteoidetes* ratio in T2D subjects after intervention with sardines (3 g EPA/DHA) [43], indicating a beneficial effect of fish on gut microbiota even though they did not find any changes in inflammation. The role of dietary fat in gut microbiota and inflammation in humans remains unresolved.

### 3.3. Other Dietary Factors

A Mediterranean diet is recommended due to its cardioprotective effect, with olives and olive oil suggested as key elements of the diet [46]. Accardi et al. investigated the effect of green olives on inflammation in 25 healthy subjects. Intervention with olives reduced plasma IL-6 levels, but no difference was observed in the gut bacteria, *Lactobacilli* [46] (Table 2). Despite evidence that red wine (RW) intake changes the composition of microbiota [63], a sub-study with 10 middle-aged men did not observe any significant differences in LPS or LBP concentration between chronic consumption of RW, dealcoholized red wine (DRW), or gin for 20 days [45]. However, changes in *Firmicutes* and *Bacteroidetes* significantly differed among the interventions. Moreover, *Prevotella* amounts were significantly increased by RW compared with DRW and gin, while RW and DRW significantly increased *Bifidobacterium* compared with gin. Both *Prevotella* and *Bifidobacterium* negatively correlated with LPS concentrations. In the post-study with a fat overload (50 g) given to five men, no differences in postprandial serum LPS, LBP, or chylomicron LPS concentration among the RW, DRW, or gin intake groups given a fatty meal were observed [45] (Table 2). Some studies have shown that apples may have a cholesterol-lowering effect and hence be cardioprotective [64]. Apples have a high content of vitamins and polyphenols, but they also contain both insoluble and soluble fiber [64]. In a four-week randomized crossover study with 23 normal weight healthy adults, the effects of whole apples, apple pomace, and clear and cloudy apple juice on inflammatory markers and gut microbiota were measured [44]. They found no differences in gut microbiota or CRP after any of the interventions (Table 2).

In summary, while interventions with green olives reduced inflammatory markers, those with RW or apples did not. Moreover, only one of the studies found changes in gut microbiota [45]. Green olives, RW, and apples are quite different products, and from the studies included in this review it is not possible to conclude whether some of these dietary products are more or less pro- or anti-inflammatory due to different effects on gut microbiota.

### 3.4. Diets

Humans eat mixed diets, not single nutrients. Consequently, there has been a shift in research focus to examine the health effects of complex diets and dietary patterns instead of single nutrients. Together with industrialization, a Westernized diet has become dominant at the expense of traditional diets. Changes in dietary preferences, together with a more sedentary lifestyle, have contributed to a dramatic increase in obesity and metabolic diseases, such as CVD and T2D. The traditional Mediterranean diet has long been related to improved health, while a Westernized diet has been associated with the opposite effect. The Mediterranean diet, characterized by a high content of nuts, fruits and vegetables, fish, and unsaturated fat instead of SFA, is well known for its cardioprotective effects [65]; however, its effect on gut microbiota is not well known.

Cotillard et al. investigated the relationships between an energy-restricted, high-protein diet and gut microbiota and CRP levels for six weeks in 49 obese or overweight subjects [47]. A six-week diet-induced weight loss, followed by a six-week weight stabilization intervention, was performed in 38 obese and 11 overweight healthy individuals. There was a significant reduction in IL-6 and CRP after 12 weeks of intervention. This reduction was most probably caused by the loss of body fat mass due to the 35% decrease in energy intake during the first six weeks of the intervention. When they divided the group based on gut microbiota composition using bacterial gene number, they observed that those with a low microbial gene count (LGC) (40% of the individuals) had more pronounced dysmetabolism and a higher CRP after six weeks of weight reduction. Gene richness increased significantly in the LGC group after the energy-restricted diet, while no change was seen in the high gene count (HGC) group (Table 2). The increase in gene richness after six weeks of an energy-restricted diet tended towards a decrease in CRP. This study indicates that diet can influence microbial richness, which could improve metabolic health [47]. In another weight reducing study, 15 overweight/obese patients with non-alcoholic fatty liver disease (NAFLD) followed a hypocaloric hyperproteic diet (HHD) for three weeks [49]. The study was designed without a control group. From baseline to after intervention, HHD significantly decreased CRP, while there were no changes in IL-6, IL-10, or TNFα. Although there were no changes in *Firmicutes* and *Bacteroidetes*, a decrease in *Lachnospira* and increase in *Blautia* and *Butyricicoccus* was observed. In addition, changes in several operational taxonomic units (OTUs) of *Bacteroidales* and *Clostridiales* were observed [49] (Table 3). The study by Umoh and co-workers investigated how Mediterranean and Healthy Eating diets affected plasma cytokines (IL-1β, IL-6, IL-8, IFNγ, TNFα, IL-4, IL-10, and IL-13), LBP and branched-chain fatty acids in a RCT with 120 healthy subjects [48]. There was a significant decrease in bacterial branched-chain fatty acids within both groups, but no change in plasma cytokines or LBP were observed [48] (Table 2). In a pilot study, eight Crohn’s patients were given a Mediterranean inspired diet (salmon, avocados, kumara, vegetables, gluten-free bread, extra virgin olive oil, green tea, honey, and fish oil capsules) for six weeks, but no significant changes in CRP or microbiota were observed after the intervention [50] (Table 3). This study was also designed without a control group, and the number of study subjects was probably too low to be able to detect a significant effect.

In the present review, two studies with calorie-restricted diets and two Mediterranean diets were included. Three of the four studies found changes in microbiota. Others have shown that a Mediterranean diet is related to changes in microbiota, with increased levels of fecal SCFA, *Bifidobacteria*, *lactobacilli*, *Eubacteria*, *Bacteroides*, and *Prevotella* [66]. However, no effect on inflammatory markers was found after intervention in either of the two Mediterranean diet studies. The study by Umoh and co-workers compared the effects of the Mediterranean diet and a healthy eating diet; the lack of effect on inflammatory markers between the groups may have been due to the fact that both were considered healthy diets. Therefore, more randomized controlled trials with healthy dietary patterns are necessary to determine if there is an effect on gut microbiota and inflammation. Obesity is characterized by a chronic low-grade inflammatory state, which will improve with weight loss [67]. Both calorie-restricted diets found reduced CRP levels after weight loss. One of the studies in this review investigated calorie restriction on gut microbiota and inflammation in patients with NAFLD [49]. They found a reduction in CRP, decreased *Lachnospira*, and increased *Blautia* and *Butyricicoccus*. Several studies have shown an association between gut microbiota, gut barrier function, and NAFLD [68]. Thus, it is of great interest to better understand how diet-induced weight loss may influence gut microbiota and the treatment of diseases like NAFLD.

## 4. Conclusions

Based on the studies included in this review, different dietary components, such as fiber and fat, may change the gut microbiota and inflammatory markers. It is plausible that the gut microbiota may affect the risk of cardiometabolic diseases, and that dietary manipulation of the microbiota might have a therapeutic benefit. However, several factors influence the bacterial composition, so the gut microbiota profile is host-specific in humans and determining its role in diseases is very challenging. In addition to the diet‒gut microbiota interaction, many factors influence human health and the risk of cardiometabolic disorders, including age, heredity, smoking, and physical activity. Furthermore, both the gut microbiome and the human genome are affected by these factors, and a complex interplay exists between human genes and the gut microbiome that is not well understood. The heterogeneity among the interventional studies discussed in this review, including the characteristics and metabolic health of participants, intervention diet, inflammatory markers, and methods for measuring and reporting changes in gut microbiota, such as information on the taxonomic levels that were analyzed, make it difficult to draw firm conclusions about the therapeutic benefits of diet, gut microbiota, and inflammation in cardiometabolic disorders. As reflected in our literature search, most studies have been performed in animals and only a small number of human intervention studies exist. More randomized controlled studies, designed with gut microbiota and inflammation as primary outcomes to ensure that enough subjects are included in the trials, are needed to fully understand the interactions between diet, gut microbiota, and systemic inflammation, and hence the risk of cardiometabolic diseases.

## Figures and Tables

**Table 1:** Examples of taxonomic levels of human gut microbiota [4,11].

Taxonomic Levels of Bacteria									
Domain/Kingdom	Bacteria								
Phylum	*Firmicutes*							*Bacteroidetes*	
Class	*Clostridia*				*Bacilli*			*Bacteroidetes*	
order	*Clostridiales*				*Lactobacilliales*			*Bacteroidales*	
family	*Clostridiaceae*		*Lachnospiraceae*		*Lactobacillacea*	*Streptococcaceae*		*Prevotellaceae*	*Bacteroidaceae*
genus	*Clostridium*	*Dorea*	*Lachnospira*	*Roseburia*	*Lactobacillus*	*Streptococcus*	*Enterococcus*	*Prevotella*	*Bacteroides*

**Table 2:** Dietary intervention studies evaluating changes in gut microbiota composition and inflammatory markers in healthy lean and overweight/obese children and adults.

Study	Population	Time of Intervention	Treatment	Changes in Gut Microbiota *	Changes in Inflammatory Markers *
**Dietary fiber and wholegrain**					
Vanegas SM et al.; AJCN, 2017, USA	*n* = 81, M/F, RG gr: 54 year, BMI 26 WG gr: 55 year, BMI 26	6 weeks	(1) Recommendations + Refined grain diet (8 g/1000 kcal) (C) (2) Recommendations + Wholegrain diet (16 g/1000 kcal)	↑ *Lachnospira* ↓ *Enterobacteriaceae* ↔ *Firmicutes*, *Bacteroidetes*, *Proteobacteria*, *Actinobacteria*, *Tenericutes*, *Verrucomicrobia*, *Cyanobacteria*, *Fusobacteria*, *Lentisphaerae*, *Roseburia* Within groups: RG: ↓ Total SCFA, acetate, propionate WG: ↓ Total SCFA, propionate	↔ TNFα, IL-8, IL-6, IL-1β, LBP ↑ TNFα (LPS stimulated blood) ↔ IFNγ, IL-8, IL-10, IL-6, IL-1β (LPS stimulated blood) ↔ IFNγ, IL-17, TNFα, IL-6, TGF-β (feces)
Morales P et al.; Clin Transl Gastroenterol, 2016, Chile	*n* = 41, M/F, C: 24 year, BMI 22.2 P: 24 year, BMI 23.1 O: 26.5 year, BMI 23.0 OP: 25.5 year, BMI 22.2	1 week	A fat-standardized diet (60 g/day) plus one of the following: (1) Maltodextrin (2 × 120 mg maltodextrin+ 16 g maltodextrin) (C) (2) Prebiotic (2 × 120 mg maltodextrin + 16 g oligofructose) (P) (3) Orlistat (2 × 120 mg Orlistat + 16 g maltodextrin) (O) (4) Orlistat/Prebiotic (2 × 120 mg Orlistat + 16 g oligofructose) (OP)	↔ Gut barrier function ↔ *Cyanobacteria*, *Actinobacteria*, *Erysipelotrichaceae*, *Bifidobacteroaceae*, *Tissierellaceae*, *Enterobacteriaceae*, *Barnesiellaceae*, *Verrucomicrobiaceae* ↔ SCFA (feces) Within groups: OP: ↑ *Bifidobacterium*, OP: ↓ Isobutyrate, isovalerate (feces)	↔ hsCRP, IL-6 ↔ Calprotectin Within groups: OP: ↓ hsCRP C, P, and O: ↓ IL-6 O: ↑ Calprotectin P and OP: ↓ Calprotectin
Nicolucci A et al. Gastroenterology, 2017, Canada	*n* = 42, M/F, C: 10.2 year, BMI 26.9 OI: 10.4 y, BMI 26.3	16 weeks	(1) Maltodextrin (3.3 g/day) (isocaloric) (C) (2) Oligofructose-enriched inulin (OI) (8 g/day)	↑ *Bifidobacterium spp.* Within groups: OI: ↑ *Actinobacteria*, ↑ *Bifidobacterium*, *Collinsella*, ↓ *Ruminococcus* C: ↓ *Clostridium XVIII*, *Actinomyces*, *Dorea*, *Eggerthella*	↔ CRP, IFNγ, IL-10, IL-1b, TNFα, IL-4, IL-33, MCP-1, LPS ↓ IL-6
Nilsson A, et al.; Clin Nutr ESPEN, 2016, Sweden	*n* = 21, M/F, 23.9 year, BMI 22.6	3 × 14 days (crossover)	(1) White wheat flour bread (WWB-ref) (148 g/day) (C) (2) Barley kernel bread without probiotics (BB) (256 g/day) (3) Barley kernel bread with probiotics (BB-pro) (256 g/day) Post-study: breakfast with WWB	↑ Breath H_2_ after BB and BB-pro groups	↔ CRP, IL-6, IL-18 ↑ s-PAI-1 after BB-pro
**Other dietary components**					
Ravn-Haren G et al.; EJN, 2013, Germany	*n* = 23, M/F, 36.2 year, BMI 22.3	5 × 4 weeks (crossover)	(1) No supplement (C) (2) Whole apples (550 g/day) (3) Apple pomace (22 g/day) (4) Clear apple juice (500 mL/day) (5) Cloudy apple juice (500 mL/day)	↔ *Bifidobacterium adolescentis*, *Bifidobacterium pseudocatenulatum*, *Bifidobacterium bifidum*, *Clostridium* clusters XI and XVIa, *Bacteroides*	↔ hsCRP
Clemente-Postigo M et al.; AJCN, 2013, Spain	*n* = 10, M, 48 year, BMI 27.6 Post-study fat overload: *n* = 5, M, 41.8 year, BMI 28.4	3 × 20 days (crossover)	(1) Red wine (RW) (272 mL/day) (2) Dealcoholized red wine (DRW) (272 mL/day) (3) Gin (100 mL/day) Post-study fat overload challenge: (1) 50 g FO (2) 50 g FO +RW (272 mL) (3) 50 g FO + DRW (272 mL) (4) 50 g FO + gin (100 mL)	↑ *Firmicutes*, *Bacteoidetes* after RW compared to DRW and gin ↓ *Firmicutes*, *Bacteoidetes* after gin compared to RW and DRW ↑ *Prevotella* after RW compared to DRW and gin ↑ *Bifidobacterium* after RW and DRW compared to gin	↔ LPS and LBP ↔ LPS and LBP post-study fat overload
Accardi G et al.; Immun Ageing, 2016, Italy	*n* = 25, M/F, 38,3 year, BMI 24.4	30 days	(1) No control group (2) Table green olives (12 olives/d)	↔ *Lactobacilli*	↓ IL-6
**Diets**					
Cotillard A et al. Nature, 2013, France	*n* = 49, M/F, - year, BMI 33.2	12 weeks	No control group Period 1: Energy-restricted, high-protein diet (6 weeks) Period 2: Weight maintenance diet (6 weeks)	LGC: ↑ gene richness at 6 weeks, and remained higher than baseline at week 12 HGC: ↔ gene richness at 6 week	↓ CRP 12 weeks ↓ IL-6 12 weeks Split the group to LGC (40%) and HGC (60%): LGC: ↑ CRP at 6 weeks compared to HGC
Umoh FI et al.; EJN, 2016, US	*n* = 120, M/F, healthy, 52 year, BMI 27.4	6 months	(1) Healthy eating diet (C) (2) Mediterranean diet	Within groups: ↓ Branched-chain bacterial fatty acids within both groups	↔ LBP, IL-1β, IL-6, IL-8, IFNγ, TNFα, IL-4, IL-10, IL-13

* Results are shown as significant (*p* ≤ 0.05) differences (↑: increase; ↓: decrease; ↔: no change) between intervention group(s) and control group, if not otherwise stated, in which (1): control group; ≥ (2): Intervention group(s). All inflammatory markers and SCFA are measured in blood and bacteria in feces, if not otherwise stated. BB: barley kernel bread; BB-pro: barley kernel bread with probiotics; BMI: body mass index; C: control; CRP: C-reactive protein; DRW: dealcoholized red wine; F: female; FO: fat overload; HGC: high gene count; hsCRP: hight sensitivity C-reactive protein; IFNγ: Interferon gamma; IL: interleukin; LBP: LPS-binding protein; LGC: low gene count; LPS: lipopolysaccharide; M: male; MCP-1: monocyte chemoattractant protein-1; O: orlistat; OI: oligofructose-enriched inulin; OP: orlistat/prebiotics, P: prebiotics; PAI-1: plasminogen activator inhibitor-1; RG: refined grain; RW: red wine; SCFA: schort-chain fatty acid; TGFβ: transforming growth factor beta; TNFα: tumor necrosis factor alpha; WG: whole grain; WWB-ref: white wheat flour bread-reference.

**Table 3:** Dietary intervention studies evaluating changes in gut microbiota composition and inflammatory markers in children and adults with chronic diseases or metabolic abnormalities.

Study	Population	Duration	Treatment	Changes in Gut Microbiota *	Changes in Inflammatory Markers *
**Dietary fiber and wholegrain**					
Benjamin JL et al.; Gut, 2011, UK	*n* = 103, M/F, Crohn’s disease, C: 39 year FOS: 40 year BMI -	4 weeks	(1) Maltodextrin (15g/day) (C) (2) FOS (15g/day)	↔ *Bifidobacteria*, *F. prausnitzii*	↓ IL-6 (dendritic cells) ↔ IL-12p40 (dendritic cells) Within groups: FOS: ↑ IL-10 (dendritic cells)
Vulevic J et al.; JN, 2013, UK	*n* = 45, M/F, ≥3 risk factors of MetS M: 42.8 year, BMI 30.7 F: 46.4 year, BMI 32.1	12 weeks	(1) Maltodextrin (5.5g/day) (C) (2) B-GOS (5.5g/day)	↑ *Bifidobacteria* ↓ *Bacteroides spp.*; *C. histolyticum group*, *Desulfovibrio spp.* ↔ Total bacteria, *Lactobacillus/Enterococcus spp.*; *Clostridium coccoides/Eubacterium rectale* group, *Atopobium* cluster, *E. cylindroides*, *E. hallii*, *β-Proteobacteria*, *Clostridium* cluster IX, *F. prausnitzii* cluster	↓ CRP, calprotectin ↔ G-CSF, IL-6, IL-10, IL-8, TNFα
Vetrani C et al.; Nutrition. 2016, Italy	*n* = 54, M/F, MetS C: 58.4 year, BMI 31.5 Wholegrain: 57.2 year, BMI 32.1	12 weeks	(1) Refined cereal goods (C) (2) Whole-grain cereal products	↑ Propionate	↔ hsCRP, TNFα, IL-1RA, IL-6
Pedersen C, et al.; Br J Nutr.; 2016, UK	*n* = 29, M, T2D, C: 58.1 year, BMI 28.4, GOS: 56.7, BMI 28.0	12 weeks	(1) Maltodextrin (5.5 g/day) (C) (2) GOS mixture (5.5 g/day)	↔ Bacterial abundance, diversity and richness Within groups: ↔ Total bacteria, *Lactobacillus Roseburia*, *Enteroacteriaceae*, *Clostridium leptum* or *Clostridium coccoides* groups.	↔ LPS, LBP, sCD14, hsCRP, IL-6, TNFα
Canfora EE et al. Gastroenterology, 2017, Nederlands	*n* = 44, M/F Prediabetic, C: 58.4 year, BMI 32.3 GOS: 59.2, BMI 33.3	12 weeks	(1) Maltodextrin (15g/day) (C) (2) GOS (15 g/day)	↔ Microbial richness and diversity ↑ *Bifidobacterium species*, *Prevotella oralis et rel.*; *Prevotella melaninogenica et rel*, *Bacteroides stercoris et rel and Sutterella wadsworthia et rel* ↔ Acetate, propionate, butyrate ↔ Acetate, propionate, butyrate (feces)	↔ IL-6, IL-8, TNF-α, LBP
**Dietary fat**					
Martin-Pelaez S et al.; Nutrients, 2016, Spain	*n* = 10, M/F, hypercholesterolemia, 35–80 year, VOO BMI 28.6 FVOO BMI 28.3, FVOOT BMI 28.4	3 weeks (crossover)	(1) OO with 80 mg PC/kg (VOO) (25 mL/day) (2) OO with 500 mg PC/kg from OO (FVOO) (25 mL/day) (3) OO with a mixture of 500 mg PC/kg from OO and thyme (1:1, FVOOT) (25 mL/day)	(1) compared to (2): ↓ Total bacteria (1) compared to (3), and (2) to (3): ↔ ↔ *Firmicutes/Bacteroidetes* ratios ↔ IgA-coated bacteria or IgA (feces) Within groups: (1) ↓ Total bacteria (2) ↑ Ig-A coated bacteria	↔ TNFα (feces), IL-6 (feces) ↔ calprotectin (feces) ↑ CRP in (2) compared to (1) and (3)
Balfego M et al.; Lipids Health Dis, 2016, Spain	*n* = 35, M/F, T2D, C: 61.2 year, BMI 28.8, Sardine: 60.0 yrear, BMI 30.5	6 months	(1) Standard diet (C) (2) Standard diet with 100 g sardines 5 days a week	↔ *Firmicutes*, *Bacteroidetes*, *Firmicutes/Bacteroidetes ratio*, *Bacteroides‒Prevotella group*, *E. rectale‒C. coccoides group*, *F. prausnitzii*, *E.coli* Within groups: ↓ *Firmicutes*, ↑ *E.coli* in (1) and (2) ↓ *Firmicutes/bacteroidetes* ratio, ↑ *Bacteroides‒Prevotella* group in (2)	↔ CRP, TNFα, IL-6, IL-10 Within groups: ↑ TNFα in group (1)
**Diets**					
Pataky Z et al.; Dig Dis Sci, 2016, Switzerland	*n* = 15, M/F, NAFLD, 50 year, BMI 34.6	3 weeks	(1) No control group (2) Eurodiet (4 products/d: breakfast, one meal, two snacks) (Hypocaloric hyperproteic diet)	↔ *Firmicutes*, *Bacteroidetes* ↓ *Lachnospira* ↑ *Blautia*, *Butyricicoccus*	↓ CRP ↔ IL-6, IL-10, TNFα
Marlow G et al.; Human Genomics, 2013, New Zealand	*n* = 8, M/F, Crohn’s disease, 45.4 year, BMI nd	6 weeks	(1) No control group (2) Mediterranean-inspired anti-inflammatory diet	↔ *Enterobacteriaceae*, *Enterococcales*, *Streptococcaceae*, *Fusobacterium*, *Bacillaceae*, *Akkermansia*, *Actinobacteria*, *Lactobacillaceae*, *Clostridium clusters* (XI, III, IX, IV, XIVa), *Bacteroides/Prevotella*	↔ CRP

* Results are shown as significant (*p* ≤ 0.05) differences (↑: increase; ↓: decrease; ↔: no change) between intervention group(s) and control group, if not otherwise stated, in which (1): control group; ≥ (2): intervention group(s). All inflammatory markers and SCFA are measured in blood and bacteria in feces, if not otherwise stated. GOS: galactooligosaccaride; BMI: body mass index; C: control; CRP: C-reactive protein; DRW: dealcoholized red wine; F: female; FOS: fructooligosaccaride; FVOO: phenolic compounds enriched virgin olive oil; FVOOT: phenolic compounds enriched virgin olive oil and thyme; G-CSF: granulocytes colony-stimulating factor; hsCRP: high sensitivity C-reactive protein; IL: interleukin; LBP: LPS-binding protein; LPS: lipopolysaccharide; M: male; MetS: metabolic syndrome; NAFLD: non-alcoholic fatty liver disease; nd: no data; OO: olive oil; PC: phenolic compond; RW: red wine; SCFA: short-chain fatty acid: T2D: type 2 diabetes; TNFα: tumor necrosis alpha; VOO: virgin olive oil.
